# Multi-drug resistance and high mortality associated with community-acquired bloodstream infections in children in conflict-affected northwest Nigeria

**DOI:** 10.1038/s41598-021-00149-1

**Published:** 2021-10-21

**Authors:** Frederick Chukwumeze, Annick Lenglet, Ruth Olubiyo, Abdulhakeem Mohammed Lawal, Bukola Oluyide, Gbemisola Oloruntuyi, Cono Ariti, Diana Gomez, Harriet Roggeveen, Chijioke Nwankwo, Nwogu Ahamba Augustine, Abiodun Egwuenu, Guy Maloba, Mark Sherlock, Shoaib Muhammad, Heiman Wertheim, Joost Hopman, Kate Clezy

**Affiliations:** 1 Médecins Sans Frontières, Anka, Zamfara Nigeria; 2 Médecins Sans Frontières, Plantage Middenlaan 14, 1018DD Amsterdam, The Netherlands; 3 Department of Clinical Microbiology, Radboudumc, Nijmegen, The Netherlands; 4 Médecins Sans Frontières, Sokoto, Sokoto Nigeria; 5 Médecins Sans Frontières, Abuja, Nigeria; 6 Cardiff University, School of Medicine, Cardiff, UK; 7 Anka General Hospital, Anka, Zamfara Nigeria; 8 Nigerian Center for Disease Control, Abuja, Nigeria; 9 Department of Patient Safety and Quality, Radboudumc, Nijmegen, The Netherlands

**Keywords:** Epidemiology, Antimicrobial resistance

## Abstract

Pediatric community-acquired bloodstream infections (CA-BSIs) in sub Saharan African humanitarian contexts are rarely documented. Effective treatment of these infections is additionally complicated by increasing rates of antimicrobial resistance. We describe the findings from epidemiological and microbiological surveillance implemented in pediatric patients with suspected CA-BSIs presenting for care at a secondary hospital in the conflict affected area of Zamfara state, Nigeria. Any child (> 2 months of age) presenting to Anka General Hospital from November 2018 to August 2020 with clinical severe sepsis at admission had clinical and epidemiological information and a blood culture collected at admission. Bacterial isolates were tested for antibiotic susceptibility. We calculated frequencies of epidemiological, microbiological and clinical parameters. We explored risk factors for death amongst severe sepsis cases using univariable and multivariable Poisson regression, adjusting for time between admission and hospital exit. We included 234 severe sepsis patients with 195 blood culture results. There were 39 positive blood cultures. Of the bacterial isolates, 14 were Gram positive and 18 were Gram negative; 5 were resistant to empiric antibiotics: methicillin-resistant *Staphylococcus aureus* (MRSA; n = 2) and Extended Spectrum Beta-Lactamase positive enterobacterales (n = 3). We identified no significant association between sex, age-group, ward, CA-BSI, appropriate intravenous antibiotic, malaria positivity at admission, suspected focus of sepsis, clinical severity and death in the multivariable regression. There is an urgent need for access to good clinical microbiological services, including point of care methods, and awareness and practice around rational antibiotic in healthcare staff in humanitarian settings to reduce morbidity and mortality from sepsis in children.

## Introduction

Sepsis, a life threatening multi-organ dysfunction caused by a dysregulated host response to infection^1^, is considered one of the main causes of mortality in children globally. In 2017, there were an estimated 20.3 million sepsis cases globally among children younger than 5 years of age^2^. Mortality from sepsis in children is also high, and up to 25% hospital mortality has been reported in a multi-center study for children admitted for intensive care with severe sepsis^3^.

Approximately 70% of all sepsis cases are thought to be community-acquired infections^4^. Public health prevention of these infections can be achieved by high vaccination coverage and improved access to adequate sanitation and water (both quality and quantity)^5^. However, early diagnosis and appropriate clinical management (antimicrobials and fluid resuscitation) reduce mortality and long-term morbidity^5^. The majority of pediatric community-acquired sepsis are from pneumonia or meningitis, but unknown sources of infection are also common^3^. Identifying a causative pathogen in patients with sepsis is also not guaranteed, even in high resource settings^6,7^. Two recent studies in pediatric intensive care units (PICUs) in seven European countries and the United States, isolated a bacteria in 54% and 48% of pediatric sepsis cases, respectively^6,7^.

The incidence and clinical outcomes of bloodstream infections (BSIs) in pediatric patients in sub Saharan Africa are poorly documented^8^. Information from humanitarian settings in Africa is even scarcer. A recent systematic review on community-acquired BSIs (CA-BSIs) in low- and middle-income countries identified only nine studies on the topic in four African countries (Kenya, Mozambique, Nigeria and Zimbabwe)^9^. A recent study in South Africa showed that 35% of BSIs identified in hospitalized children were community-acquired infections (the remainder healthcare associated or hospital acquired), with 47% due to Gram-negative bacteria (GNB) infections (mostly *E. coli*) and 50% due to Gram-positive bacterial (GPB) infections (mostly *Staphylococcus aureus* and *Streptococcus pneumoniae*)^8^. Another study in South Africa with CA-BSI surveillance in pediatric inpatients, showed that 40% of infections were due to GNB (54% were *E. coli*) and 60% due to GPB (45% from *S. aureus*)^10^.

Surveillance for antibiotic resistance is improving across the continent, but information on bacterial susceptibility to commonly used antibiotics for the treatment of BSIs in pediatric patients remains limited and reported in non-standardized ways^11^. Even so, reported resistance to commonly prescribed antibiotics across Africa is high^12^ and it is assumed to be similar in humanitarian contexts. A systematic review on resistance in bacteria isolated in children with sepsis in Africa showed that *E. coli* isolates were resistant to ampicillin, gentamicin and ceftriaxone of 93%, 29% and 16% respectively^12^. Similarly, *S. aureus* isolates showed resistance of 90%, 29% and 20% for ampicillin, gentamicin and cloxacillin respectively^12^.

**Figure 1:**
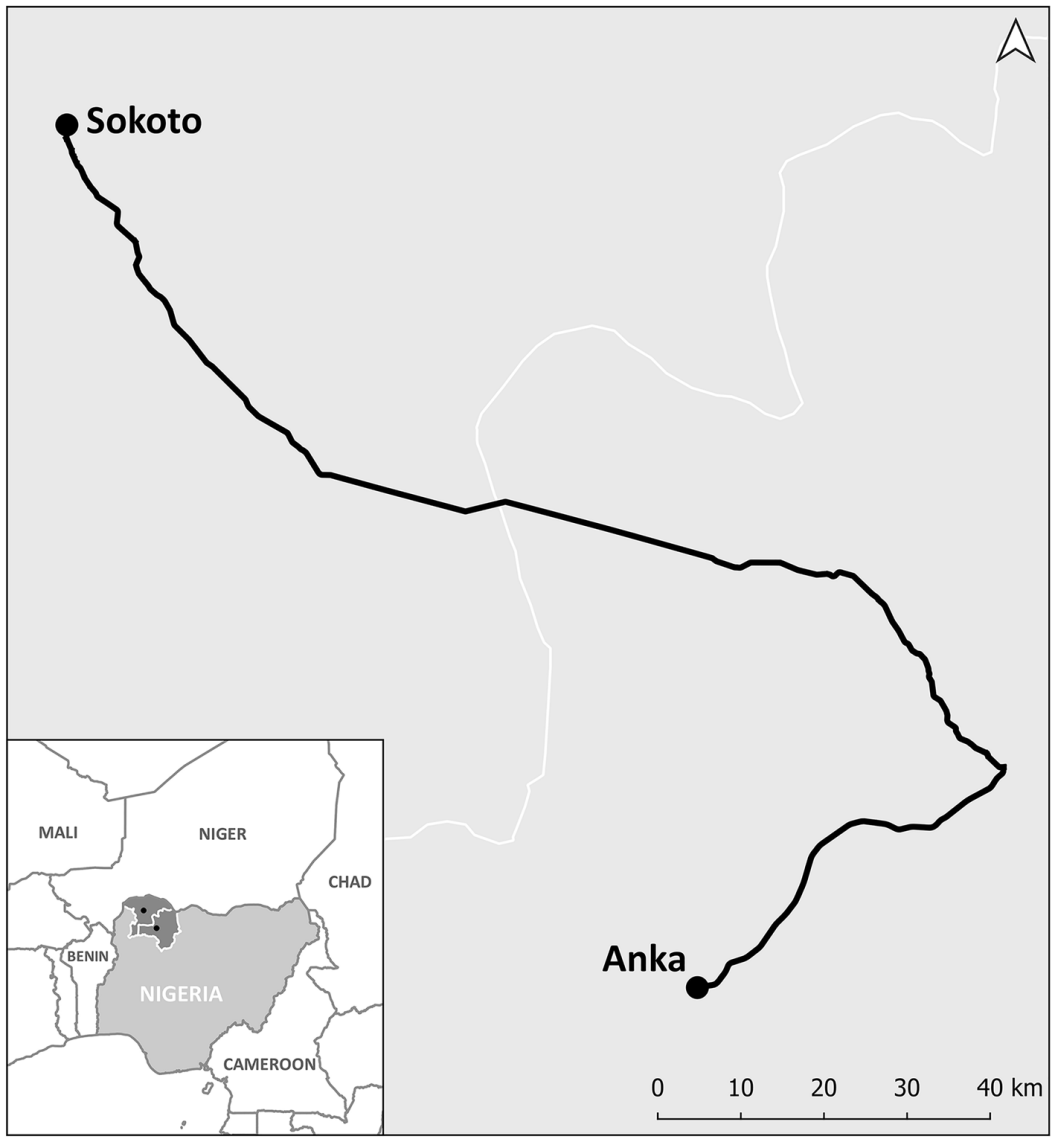
Map indicating the location of Anka (Zamfara State) where AGH is and Sokoto (Sokoto State) where the microbiology laboratory is based with the road that connects them. Note: Contains information from OpenStreetMap and OpenStreetMap Foundation, which is made available under the Open Database License.

Since 2014 Zamfara state in northwest Nigeria is affected by an intensified internal conflict and population displacement^13^. Since 2018, the Ministry of Health and Médecins Sans Frontières (MSF) implement antimicrobial resistance (AMR) control activities (antibiotic stewardship, infection prevention and control and microbiological surveillance) in the pediatric inpatient, isolation and inpatient therapeutic feeding centre (ITFC; dedicated to children with severe acute malnutrition) of Anka General Hospital (AGH) (Fig. 1). We report the clinical, epidemiological and microbiological findings from pediatric admissions to the hospital between November 2018 and August 2020 with suspected severe sepsis.

## Methods

### Study setting

Access to the hospital is hampered by the lack of primary healthcare referral options, distance and security to travel. A hospital emergency triage functions as the sole point for hospital admission to the isolation, inpatient or ITFC wards (150 beds total). The hospital discharged an average of 769 pediatric patients per month during 2019 (range: 419–1066 per month; unpublished data, MSF).

### Severe sepsis upon admission management

From October 2018, we established surveillance for CA-BSIs in pediatric patients that presented to the emergency triage with signs and symptoms of severe sepsis. A case of suspected severe sepsis was any pediatric patient over the age of 2 months that presented with at least two of the following clinical criteria: axillary temperature > 38 °C or < 35.5 °C, tachycardia or bradycardia for their age, tachypnea for age and/or oxygen saturation < 92% or leukocytosis or leukopenia or > 10% immature neutrophils and one of the following criteria: decreased level of consciousness (pain or unresponsive on the alert/verbal/pain/unresponsive [AVPU] scale) or circulatory insufficiency (defined as tachycardia with weak pulse or capillary refill time > 3 s or oligo-anuria).

Any severe sepsis patient had a blood culture taken and was treated using standard clinical protocols^14^. Empirical treatment for severe sepsis is with intravenous (IV) ceftriaxone monotherapy with the addition of: cloxacillin (suspected sepsis source: skin/soft tissue); metronidazole (suspected sepsis source: aspiration pneumonia); clindamycin (suspected sepsis source: bone/joint); metronidazole (suspected sepsis source: intestinal/biliary or abdominal).

We collected information on: age, sex, weight, date of admission, date of and reason for exit, primary diagnosis at exit, admission ward, suspected source of sepsis at admission, signs of sepsis at admission (respiratory rate, heartrate, skin color, urine output etc.), malaria positivity from rapid diagnostic tests, use of antibiotics prior to admission to hospital, start and end dates of IV antibiotics received during hospitalization and clinical outcome.

### Microbiological testing

Blood cultures were collected from severe sepsis patients identified at admission using an aseptic technique. Not all patients presenting with severe sepsis had blood cultures performed due to transport problems, staff availability and logistical constraints (i.e. security restrictions for transport, temporary rupture of materials etc.). For infants or malnourished children, a single blood volume of 1-2 mL was collected and for pediatric patients > 1 years of age a single blood volume of 2.5–5 mL was collected using neonatal and pediatric aerobic blood collection bottles respectively (Liofilchem, Italy). Following collection, culture bottles were incubated at 35 °C. Culture bottles were transported at ambient temperature in triple packaging by a private taxi service to the microbiology laboratory (established in July 2018) in Noma Children’s Hospital (NCH) in Sokoto (Sokoto State) (3 h drive from Anka). Positive blood cultures were Gram stained to determine subsequent choice of enriched and/or selective media for subculture (Polyvitex (PVX), Columbia Colistin Nalidixic Acid (CNA), MacConkey (MAC) or CHROMagar Orientation (CRO) agar plates). Colonies obtained from the media were identified using standard biochemical tests and the API^®^ system (bioMérieux, France). Antibiotic susceptibility was assessed using the Kirby–Bauer disk diffusion method on Mueller–Hinton agar and interpreted following the European Committee on Antimicrobial Susceptibility Testing (EUCAST) recommended breakpoints from 2019^15^. The control organisms used for quality control were American type culture collection strains (ATCC) based on the EUCAST recommendation.

A confirmed CA-BSI was defined as any patient for whom a positive blood culture (bacterial or yeast) was obtained that was not a contaminant. Coagulase-negative Staphylococci, *Bacillus* sp., *Corynebacterium* sp. or *Propionibacterium* sp. were considered contaminants. For other bacteria or yeast, the laboratory team discussed the result with the antibiotic stewardship focal point in AGH to determine if the organism was the likely cause of symptoms. If the microorganism was determined not to fit the clinical presentation, the bacteria were considered a probable contaminant.

### Data sources

We combined data from three separate sources for this current analysis: the health information data (District Health Information Software [DHIS2]), a severe sepsis database (Epi Data Manager; http://www.epidata.dk/download.php; which stored the additional clinical and epidemiological data collected for all pediatric patients with severe sepsis) and the WHONET microbiological database (http://www.whonet.org).

### Data analysis

We merged all datasets and retained data from DHIS2 and WHONET if the patient identification number was available in the severe sepsis dataset. Patient characteristics at admission were described for all severe sepsis cases by age group, sex, ward of admission, duration of hospitalization and clinical outcome. We were unable to calculate a Pediatric Early Warning Score (PEWS) from the data available, thus we created a modified severity score. We assigned scores of 0, 1, 2 or 3 (3 being the most severe) to each category of presentation for capillary refill time (< 3 s = 0; CRT 3–4 s = 1 and CRT > = 5 s = 2), oxygen saturation (95–100% = 0; 90–95% = 1; < 90% = 2; < 80% = 3), AVPU (Alert, Verbal, Pain, Unresponsive: A = 1, V = 2, P = 3, U = 3), respiratory rate (normal = 0, tachypnoea = 2, bradypnea = 3), respiratory distress (1), tachycardia (1) and bradycardia (1). We calculated the 25th quartile and the median for the total severity scores in this patient population. Low severity was defined as being below the 25% quartile, moderate severity was from the 25th–50th quartile and high severity was greater than the 50% quartile.

Due to resource constraints, we limited data collection related to treatment received during hospitalization to IV antibiotics only. Appropriateness of antibiotic therapy was independently reviewed by FC and KC using compliance to the MSF Pediatric Guidelines^14^ for diagnosis and treatment of sepsis and fever and evaluating whether antibiotics were switched when results of blood cultures were available. The clinical presentation of severe sepsis, clinical course and outcomes was compared between patients admitted to the ITFC or the pediatric/isolation wards. We assessed the differences between the proportions of outcomes in both hospital wards using Pearson’s chi-squared test. The time of IV antibiotic administration was calculated as the time from IV insertion to removal (for reasons of death, de-escalation, discharge etc.). We performed Kaplan–Meier survival analysis to assess the time to death following IV antibiotic administration in the ITFC and pediatric/isolation wards. The log rank test was used to determine if the difference between the survival curves was statistically significant.

We compared epidemiological and clinical risk factors upon admission for patients with confirmed bloodstream infections with those who had blood culture contamination or a negative blood culture using descriptive statistics and testing for significant differences using Chi-square and Fisher exact test (p-values under 0.05 were considered statistically significant). Only one clinical variable showed evidence of a statistically significant difference between the two groups, thus we did not conduct further regression analysis.

To understand the risk factors that contributed to death in the severe sepsis patient group, we calculated person-time as the days from date of admission until date of death or other outcome (discharged, Left Against Medical Advice [LAMA], Lost to Follow Up [LTFU] and referred). We were unable to correctly ascertain treatment switch for each patient (as we only collected data on IV antibiotic treatment and not oral antibiotic treatment). Time to treatment switch to appropriate antibiotics was not taken into account in this analysis. Crude and adjusted rate ratios (iRR) were calculated using Poisson regression models. All variables with p ≤ 0.2 in the univariate analysis were included in the multivariable model.

All data analysis was done using R (Version 4.0.2) and Rstudio (Version 1.3.1056). Microbiological data was analyzed using the AMR for R package which applies EUCAST clinical breakpoints and dosing of antibiotic guidance 2020^16,17^.

### Ethics approval and consent to participate

This study used routinely collected clinical and microbiological data from patients to inform their clinical management and monitor program implementation. As the data was not collected for research purposes, no explicit informed consent for the collection of data was required. The Nigerian Federal Ethical Review Board and the Zamfara Ethical Review board exempted the study from ethical review. This research fulfilled the exemption criteria set by the Médecins Sans Frontières Ethics Review Board for a posteriori analyses of routinely collected clinical data and thus did not require MSF ERB review. It was conducted with permission from Melissa McRae, Medical Director, Operational Centre Amsterdam (OCA), Médecins Sans Frontières.

## Results

### Overall characteristics of severe sepsis patients

We analysed information from 234 patients of severe sepsis who were admitted to AGH between 1 November 2018 and 31 August 2020. The sex distribution between patients was similar, most patients were below the age of 2 years and most were admitted to the pediatric ward (Table 1). Thirty-five percent (n = 82) of severe sepsis patients died during their hospitalization with almost half (49%) dying within 24 h of admission to hospital (Table 1). Most ITFC patient (91%) were younger than 2 years of age, compared to 64% in the pediatric/isolation ward (p < 0.001). ITFC patients also had a significantly higher mortality rate compared to those in the pediatric and isolation ward (49% vs. 28%) (Table 1).

**Table 1:** Epidemiological characteristics of pediatric patients admitted with severe sepsis in AGH between November 2018 and August 2020.

Characteristic	Total (n = 234)		ITFC ward (n = 77)		Pediatric and isolation ward (n = 148)		p-value
	n	%	n = 77	%	n = 148	%	
**Sex**							
Female	121	52	45	59	70		0.12*
Male	110	48	31	41	78		
Missing	3		1				
**Age (years)**							
Mean (SD^¥^)	2.14 (2.09)		1.46 (0.84)		2.49 (2.30)		< 0.001**
Median	1.75		1.33		2.00		
IQR	1.00–3.00		0.92–2.00		1.00–3.00		
**Age groups**							
0–2	165	73	70	91	95	64	< 0.001***
3–4	40	18	6	7.8	34	23	
5–9	15	6.7	1	1.3	14	9.5	
10–14	5	2.2	0	0	5	3.4	
Missing	9						
**Ward admitted to**							
Inpatient ward	139	62					
ITFC^¥¥^	77	34					
Isolation	9	4.0					
Missing	9						
**Clinical outcome**							
Discharged	129	55	32	42	91	61	0.005***
Death	82	35	38	49	41	28	
LAMA^¥¥¥^	12	5.1	2	2.6	10	6.8	
Lost to follow up	4	1.7	1	1.3	3	2.0	
Referred	7	3.0	4	5.2	3	2.0	
**Duration of hospitalisation**							
24 h or less	42	19	16	21	26	18	0.8*
24–48 h	23	10	8	11	15	10	
> 48 h	157	71	51	68	106	72	
Missing	12		2		1		
**Time from admission to death (for those that died) (n = 82)**							
24 h or less	38	49	15	41	23	56	0.3*
24–48 h	9	12	6	16	3	7.3	
> 48 h	31	40	16	43	15	37	
Missing	4		1				

^¥^*SD* standard deviation, ^¥¥^*ITFC* Inpatient Therapeutic Feeding Centre, ^¥¥¥^*LAMA* Left Against Medical Advice, *Chi-square test of independence, **Wilcoxon rank-sum test, ***Fisher's exact test.

Most patients with severe sepsis at admission had a suspected respiratory focus (38%) or an unknown focus (21%) (Table 2). Patients in the ITFC were more likely to have respiratory and gastrointestinal focus of sepsis whereas those admitted to the pediatric or isolation wards were more likely to have an unknown or suspected CNS focus (Table 2). The proportion of patients with a suspected source of sepsis from skin and soft tissue infection were similar in both wards. The malaria positivity rate in those presenting with suspected CNS focus was significantly higher compared to all the other categories of suspected focus of sepsis (82% vs. 58%, p = 0.003). Only two patients were recorded as having received antibiotics before admission to AGH, but this variable was missing for 165 severe sepsis patients (71%).

**Table 2:** Clinical characteristics at admission of pediatric patients with severe sepsis at AGH overall and by ward of admission (ITFC or pediatric/isolation ward), November 2018–August 2020.

Characteristic	Overall (n = 234)		ITFC ward (n = 77)		Pediatric and isolation (n = 148)		p-value
	n	%	n	%	n	%	
**Suspected sepsis focus**							
Respiratory tract	89	38	33	43	53	36	0.019***
Unknown	49	21	13	17	34	23	
Gastrointestinal tract	48	21	22	29	23	16	
Central nervous system	33	14	4	5.2	28	19	
Skin and soft tissue	8	3.4	3	3.9	5	3.4	
Urinary tract	3	1.3	1	1.3	2	1.4	
Bone and joint	2	0.9	1	1.3	1	0.7	
Missing	2		0		2		
**Temperature (°C)**							
Mean (SD^¥^)	38.05 (1.86)		37.96 (2.08)		38.14 (1.70)		1.0**
Median	38.60		38.65		38.65		
IQR^¥¥^	37.30–39.40		37.30–39.40		37.38–39.40		
Missing	1		1		0		
Fever	165	71	54	70	106	72	1.0*
Hypothermia	23	9.8	11	14	11	7.4	0.2*
**Respiration**							
Tachypnoea	144	62	40	52	104	70	0.01*
Bradypnea	15	6.4	7	9.1	8	5.4	0.4*
Respiratory distress	194	83	65	84	122	82	0.8*
**Oxygen saturation (%)**							
< 80	36	16	13	17	22	15	0.2*
80 to < 90	33	14	6	7.8	26	18	
90 to < 95	34	15	11	14	23	16	
95 to 100	126	55	47	61	72	50	
Missing	5		0		5		
**Urine output abnormal**							
Abnormal	57	25	21	29	35	24	0.5*
Normal	170	75	52	71	111	76	
Missing	5	4		2			
**Heart rate**							
Tachycardia	154	66	46	60	108	73	0.061*
Bradycardia	33	14	16	21	17	11	0.095*
**Pulse quality**							
Strong	63	30	14	21	47	35	0.13*
Weak	110	52	38	57	66	49	
Thready	38	18	15	22	23	17	
Missing	23	10		12			
**Oral mucosa**							
Dry and cracked	31	13	12	16	19	13	0.9*
Dry intact	176	76	56	74	111	76	
Moist	24	10	8	11	16	11	
Missing	3		1		2		
**Capillary refill time**							
> = 5 s	38	16	14	18	22	15	0.3*
3–4 s	112	48	41	53	22	15	
< 3 s	83	36	22	29	57	39	
**Temperature gradient**							
Limbs = trunk	180	79	18	24	28	19	0.5*
Limbs cooler than trunk	48	21	57	76	116	81	
Missing	6	2		4			
**Consciousness level**							
Alert	34	15	11	14	20	147	0.5*
Verbal	63	27	26	34	36	24	
Pain	102	44	29	38	68	46	
Unresponsive	34	15	11	14	23	16	
Missing	1				1		
**Severity score**							
High	60	33	23	37	37	31	0.6*
Moderate	35	19	10	16	25	21	
Low	86	48	29	47	57	48	
Missing	53	15					
Severe acute malnutrition	86	37	71	92	12	8.1	< 0.001*
Sickle cell anemia suspected	2	0.9	0	0	2	1.4	0.5***
Measles suspected	18	7.7	6	7.8	12	8.1	1.0*
Tuberculosis	1		0	0	1	0.7	1.0***
**Malaria RDT**							
Positive	143	61	45	58	93	63	0.6*
**Malaria species (available for 114 patients)**							
*P. falciparum*	60	53	22	56	38	53	0.3*
*P. falciparum* + PAN	16	14	3	7.7	13	18	
Negative	38	33	14	36	21	29	
**Antibiotics received before hospitalisation**							
Yes	2	2.9	0	0	2	4.3	0.6***
No	33	48	11	55	20	43	
Don’t know	34	49	9	45	25	53	
Missing	165	57	101				

^¥^*SD* standard deviation, ^¥¥^*IQR* inter quartile range, *Chi-square test of independence, **Wilcoxon rank-sum test, ***Characteristic Fisher's exact test.

Patients in ITFC received IV antibiotics for a median of four days compared with three days in the pediatric/isolation ward (Table 3). The main reason for cessation of IV antibiotics was death in the ITFC and discharge in the pediatric/isolation ward (Table 3). The Kaplan–Meier survival analysis showed little evidence of a difference between the time to death following IV insertion (p = 0.08; supplemental information). Most patients received IV ceftriaxone (in line with clinical algorithms). Receipt of two or more antibiotics was more common in the ITFC patients (77% vs 46.5%) as was cloxacillin use (Table 4). Fewer children in ITFC received appropriate antibiotics compared to those in admitted to pediatric/isolation wards (61% vs 78%, p = 0.01) (Table 3).

**Table 3:** Clinical course and treatment received with IV antibiotics for pediatric patients admitted with severe sepsis in AGH, November 2018-August 2020.

Characteristic	Overall (n = 234)		ITFC ward (n = 77)		Pediatric and isolation (n = 148)		p-value
	n	%	n	%	n	%	
Mean IV line duration (days, SD^¥^)	4.65 (4.37)		5.25 (4.85)		4.16 (3.28)		0.2**
Median IV line duration (IQR^¥¥^)	4 (2–6)		4 (2–6)		3 (2–6)		
**Reasons for IV removal**							
Discharged	116	50	27	35	84	57	0.004***
Died	75	32	33	43	39	26	
LAMA^¥¥¥^	14	6.0	3	3.9	11	7.4	
Thrombosis	13	5.6	7	9,1	6	4.1	
Changed to oral medication	10	4.3	6	7.8	3	2.0	
Transferred	3	1.3	1	1.3	2	1.4	
Other	3	1.3	0		3	2.0	
Missing							
**IV Antibiotics received**							
Ceftriaxone	229	98	76	99	144	97	0.7***
Cloxacillin	109	47	52	68	53	36	< 0.001*
Gentamycin	27	12	12	16	13	8.8	0.2*
Metronidazole	27	12	10	13	16	11	0.8*
Ampicillin	5	2.1	0	0	5	3.4	0.2***
Amoxiclav	3	1.3	3	3.9	0	0	0.039***
Ciprofloxacin	2	0.9	1	1.3	1	0.7	1.0***
Clindamycin	4	1.7	3	3.9	1	0.7	0.12***
Cefazolin	1	0.4	0	0	1	0.7	1.0***
**Total IV antibiotics received**							
0	2	0.9	1	1.3	1	0.7	< 0.001***
1	99	42	17	22	78	53	
2	100	43	42	55	55	37	
3	26	11	14	18	10	6.8	
4	7	3.0	3	3.9	4	2.7	
**Treatment received**							
Appropriate	169	72	47	61	116	78	0.010***
Not appropriate	53	23	26	34	24	16	
Cannot be assessed	12	5.1	4	5.2	8	5.4	

^¥^Standard Deviation, ^¥¥^Interquartile range, ^¥¥¥^Left Against Medical Advice, *Chi-square test of independence,**Wilcoxon rank-sum test, ***Fisher's exact test

**Table 4:** Blood culture results for pediatric patients admitted with severe sepsis in AGH, November 2018–August 2020.

Isolate categories	Isolate characteristics	n	%
**Suspected contaminants**			
Overall	Total	40	
	Gram positive bacteria	25	64
	Gram negative bacteria	10	28
	*Candida albicans*	1	2.5
	Yeast unknown	1	2.5
	Unknown	3	7.5
Gram positive contaminants	Coagulase-negative *Staphylococcus* (CoNS)	10	40
	Unknown gram positive	10	40
	*Corynebacterium* sp.	5	20
Gram negative contaminants	*Aeromonas hydrophila*	5	50
	*Burkholderia cepacia*	3	30
	*Achromobacter*	1	10
	*Aeromonas* sp.	1	10
**Blood stream infection**			
Overall	Total	39	
	Gram positive bacteria	14	36
	Gram negative bacteria	18	46
	Yeast	6	15
	Unknown	1	2.6
Gram positive bacteria	*Staphylococcus aureus*	11	79
	*Streptococcus dysgalactiae*	1	7.1
	*Streptococcus group B*	1	7.1
	*Streptococcus group F*	1	7.1
Gram negative bacteria	*Pseudomonas* sp.	5	28
	*Pseudomonas aeruginosa*	3	17
	*Salmonella* sp.	3	17
	*Escherichia* coli	1	5.6
	*Klebsiella pneumonia*	1	5.6
	*Proteus vulgaris*	1	5.6
	*Serratia liquefaciens*	1	5.6
	Unknown	3	17
Yeast	*Candida albicans*	1	17
	Unknown	5	83

### Confirmed bacterial bloodstream infections

195 patients with severe sepsis (83.3%) had blood cultures taken. They were negative in 116 patients (59.5%) and positive in 79 patients (40.5%). Forty isolates (50.6%) were considered contaminants and 39 isolates (49.3%) were considered confirmed CA-BSIs. Fifty percent of contaminants were coagulase-negative *Staphylococcus* and unknown GPB (Table 4). We also classified six *Aeromonas* sp. isolates, three *Burkholderia cepacia* and one *Achromobacter* sp. isolates as contaminants. Of the confirmed CA-BSIs, most were GNB (n = 18), followed by GPB (n = 14) and yeasts (n = 6). Most of the GNB were *Pseudomonas* sp. (n = 8), three of which were *P. aeruginosa* (all sensitive to gentamicin). We identified three ESBL positive isolates of *Escherichia coli*, *Klebsiella pneumoniae* and *Serratia liquefaciens*. Most of the GPB were *Staphylococcus aureus* (n = 11) of which two were methicillin resistant (MRSA). Six blood cultures contained yeast of which one was *Candida albicans* and the remainder were unspecified (Table 4).

Fifty-one percent of patients with confirmed BSIs were alert (A) and verbal (V) at admission compared to 37% of patients without confirmed BSIs. Patients with confirmed BSIs were less likely to be unresponsive at admission (2.6% vs 19%, p = 0.008). All other clinical criteria at admission were similar between these two patient groups.

Information on the time to communication of preliminary and final results was available for 151 blood cultures (77%) and 158 blood cultures (81%) respectively. Preliminary results were communicated with a mean of 3.2 days (SD: 1.16; median = 3 days) from the date of admission and final results were communicated with a mean of 7.1 days (SD: 2.4 median = 7 days) from the date of admission. For 116 (76%, n = 153) patients, the final blood culture results arrived an average 4.9 days (median: 4) after their exit from hospital (for all clinical outcomes). For 36 patients (24%), the final blood culture results arrived an average of 6.3 days (median: 5 days) before their exit from hospital. For 157 patients, for whom the information was available, 93% (n = 146) received their blood culture results after the last dose of IV antibiotics was administered.

### Risk factors for death

The univariable Poisson regression only identified male patients as having a lower risk of death compared to female patients (p = 0.04; Table 5). In the multivariable model, no further epidemiological or clinical characteristics showed a statistically significant association with death (Table 5).

**Table 5:** Mortality rate and factors associated with death in pediatric patients admitted with severe sepsis in AGH in univariable and multivariable Poisson regression, November 2018–August 2020.

Characteristic	Deaths (n)	Days In hospital	Mortality rate per 1000 person days		Univariable			Multivariable		
			Rate	CI95%*	iRR**	CI95%	p-value	aiRR***	CI95%	p-value
**Sex**^**¥**^										
Female	49	587.6	83.4	63.0–114.4						
Male	32	623.8	51.3	31.1–67.6	0.6	0.4–1.0	0.04	0.7	0.5–1.0	0.08
**Age group**^**¥**^										
0–2	62	909.9	68.1	52.7–90.2	0.7	0.4–1.6	0.2	0.6	0.3–1.3	0.06
3–4	9	232.1	38.8	23.5–82.1	0.4	0.2–1.1		0.4	0.2–0.9	
5 and over	8	85.4	93.7	32.2–162.4	Ref			Ref		
**Ward**^**¥**^										
ITFC	38	491.3	77.3	54.9–108.7	Ref		0.2	Ref		
Pediatric/isolation	41	736.1	55.7	39.9–77.2	0.7	0.5–1.2		0.8	0.5–1.3	0.4
**Malaria RDT**^**¥**^										
Negative	33	583.4	56.6	34.6–73.9						
Positive	49	644.0	76.1	57.4–105.3	1.4	0.9–2.2	0.2	1.4	1.0–2.2	0.09
**Suspected measles**										
No	74	1136.2	65.1	49.0–80.8						
Yes	8	91.2	87.7	37.1–163.5	1.4	0.6–2.8	0.3			
**Sepsis focus**										
Other	7	108.1	64.8	30.2–135.1	Ref		0.6			
CNS	6	169.0	35.5	18.3–84.0	0.5	0.2–1.7				
GI tract	18	241.5	74.5	49.2–137.0	1.1	0.5–2.8				
Respiratory	33	466.4	70.8	40.7–87.9	1.0	0.5–2.5				
Unknown	18	227.4	79.2	46.8–126.0	1.2	0.5–3.0				
**Severity**										
Low	27	456.1	59.2	42.5–87.8	Ref		0.9			
Moderate	12	196.2	61.2	37.3–109.3	1.0	0.7–2.0				
High	25	352.8	70.9	46.1–99.2	1.1	0.5–2.0				
**Confirmed BSI**										
No	66	1007.4	65.5	46.4–79.3	Ref					
Yes	16	220.0	72.7	49.0–136.5	1.1	0.6–1.9	0.8			
**Treatment received**^**¥**^										
Appropriate	54	749.9	72.0	47.9–87.1	1.4	0.9–2.5	0.2	1.5	0.9–2.3	0.1
Inappropriate	19	383.4	49.6	32.8–86.7	Ref					
Unable to be assessed	9	94.1	95.6	49.6–178.6	2.0	0.9–4.4		2.0	0.9–4.0	

^¥^Included in multivariable regression model, *95% Confidence Interval, **Incidence Rate Ratio, ***Adjusted incidence Rate Ratio

## Discussion

In this acute humanitarian setting in Zamfara state, pediatric patients that present for hospital care with signs and symptoms of severe sepsis have high mortality rates (35%) and approximately 20% have confirmed CA-BSIs. We were unable to determine any clear epidemiological or clinical risk factors for death in this patient group. We also showed that confirmed CA-BSIs are not associated with higher mortality rates compared with other causes. This might be from the high proportion of patients receiving the correct empirical antibiotic treatment upon admission.

The mortality in pediatric patients with suspected bloodstream infections in Anka is higher than the average pediatric mortality reported from secondary hospitals in Nigeria in Aba (5.7%)^18^ and Asaba (5.8%)^19^. It is also higher than the overall average monthly pediatric hospital mortality from AGH (8.3%, MSF unpublished data). This speaks to the severity of disease in patients presenting with severe sepsis at AGH. Even though global estimates for hospital mortality from sepsis in children is around 25%^3^, the higher rates in Anka might also be linked to difficulties in accessing the hospital as suggested by the high proportion of patients that die within 24 h of being admitted to the hospital^20^.

The 20% blood culture positivity rate identified in our study is similar to those obtained in the few available studies from hospitalized children < 5 years of age in Nigeria, ranging between 11 and 30%^21–23^. Half of the bacteria implicated in confirmed CA-BSI in this study were GNB. This is similar to the results from a recent meta-analysis of pediatric CA-BSI that included six studies from African hospitals where 54.8% (CI95% 45.1–64.4) of infections were due to GNB. ESBL producing bacteria were identified in 22% of the GNB isolates which is higher than the 6% of ESBL producing Enterobacterales isolated in Enugu (Nigeria) in 2019^24^ but closer to the 39% of ESBL positive GNB reported from central and northwest Nigeria in 2020 in similar patient groups^22^. It has been shown that malnourished children have an increased risk for acquisition of ESBL producing Enterobacterales when exposed to amoxicillin^25^. The high prevalence of ESBL-GNB at admission in Anka should also be a reminder for antibiotic stewardship and infection prevention and control in the hospital, due to the high numbers of severe acute malnutrition patients treated and risk for hospital-acquired infections with ESBL-GNB in this patient group. We were unable to differentiate the *Salmonella* isolates, but it has been previously described that the majority of these isolates in children in Nigeria are from *S. typhi*^21,23^. We are unable to comment on the high rate of yeast positive blood cultures in our patient population due to the limited additional clinical information available on these patients.

The widespread use of antibiotics in Nigeria without doctors prescriptions is well documented^26^. We had difficulty identifying which patients had received antibiotics and what these antibiotics were prior to their admission to hospital. This may have influenced the treatment choice for the individual patient and it could have facilitated the interpretation of negative blood cultures. We currently do not test for HIV status in the pediatric population, and even though the overall prevalence is assumed to be low, this information would allow for better interpretation of blood culture results (particularly yeast). Approximately half of the positive blood cultures were contaminated which is an issue that we are actively working to reduce through training staff in aseptic blood collection. Due to resource constraints, we have only been using a single blood culture bottle, but are working towards using two bottles as standard practice. Our ability to analyze the clinical progression of severe sepsis patients for their duration of hospitalization was limited due to the restricted amount of data we were able to collect thus we were unable to evaluate fluid resuscitation and de-escalation to oral antibiotics. This could further improve the current clinical management and AMR activities if resources allow.

## Conclusions

Pediatric sepsis accounts for approximately 8% of all critically ill children^27^. In 2020 WHO called for global action on sepsis with a focus on improving data from low-income countries with better definitions for sepsis and more information on microbiological aspects and antibiotic resistance^28,29^. Our study sheds much needed light on the incidence and causative organisms implicated in pediatric sepsis in a humanitarian context in Nigeria. Interventions around antibiotic stewardship require context-specific approaches^30^ and humanitarian settings are no different. The current study reinforces the urgent need for improved clinical bacteriology to improve awareness and practice around optimal antibiotic use in clinical care providers in these locations^31^. In the absence of microbiology, new, affordable and validated point of care diagnostic tools might also reduce inappropriate antibiotic use^32^. In addition, the mainstay activities of humanitarian response to increase vaccination coverage, improve water and sanitation conditions, increase access to early diagnostics and healthcare and improve the nutritional status of affected populations will serve to prevent CA-BSIs. These preventative measures should be included as part of context specific stewardship activities in humanitarian settings.

## Supplementary Information

Supplementary Figure S1.

## Data Availability

MSF has a managed access system for data sharing. Data are available on request in accordance with MSF’s data sharing policy. Requests for access to data should be made to data.sharing@msf.org. For more information please see: (1) MSF’s Data Sharing Policy: http://fieldresearch.msf.org/msf/handle/10144/306501, (2) MSF’s Data Sharing Policy PLOS Medicine article: http://journals.plos.org/plosmedicine/article?id=10.1371/journal.pmed.1001562.

## Abbreviations

AGH: Anka General Hospital
AMR: Antimicrobial resistance
AVPU: Alert, Verbal, Pain, Unresponsive
CA-BSI: Community acquired bloodstream infection
CAN: Columbia Colistin Nalidixic Acid
CNS: Central nervous system
CRO: CHROMagar Orientation
ESBL: Extended Spectrum Beta-Lactamase
EUCAST: European Committee on Antimicrobial Susceptibility Testing
GNB: Gram negative bacteria
GPB: Gram positive bacteria
HIV: Human immunodeficiency virus
IQR: Inter quartile range
ITFC: Inpatient therapeutic feeding centre
IV: Intravenous
LAMA: Left against medical advice
LTFU: Lost To Follow up
MAC: MacConkey
MRSA: Methicillin Resistance *Staphylococcus aureus*
MSF: Médecins Sans Frontières
NCH: Noma Children’s Hospital
PEWS: Pediatric Early Warning Score
PICU: Pediatric Intensive Care Unit
PVX: Polyvitex
RDT: Rapid Diagnostic Test
SD: Standard deviation
WHO: World Health Organisation
