# Supplementary material for: Multi-drug resistance and high mortality associated with community-acquired bloodstream infections in children in conflict-affected northwest Nigeria
Source: Sci Rep. 2021 Oct 21;11:20814. doi: 10.1038/s41598-021-00149-1 (PMC8531324; doi:10.1038/s41598-021-00149-1)

**Figure S1**: Kaplan Meijer survival curve for the time to IV removal due to death in the ITFC ward and the pediatric/isolation ward


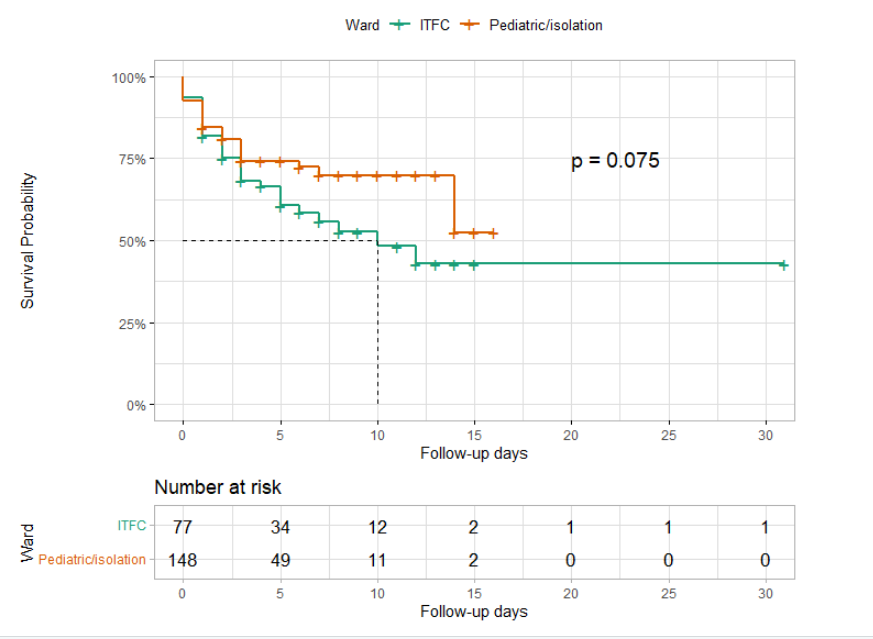

Supplement: Supplementary file 1 — Supplementary Figure S1. [file 41598_2021_149_MOESM1_ESM.docx]
